# Investigation of Naturally Occurring Single-Nucleotide Variants in Human TAAR1

**DOI:** 10.3389/fphar.2017.00807

**Published:** 2017-11-24

**Authors:** Jessica Mühlhaus, Juliane Dinter, Sabine Jyrch, Alexander Teumer, Simon F. Jacobi, Georg Homuth, Peter Kühnen, Susanna Wiegand, Annette Grüters, Henry Völzke, Klemens Raile, Gunnar Kleinau, Heiko Krude, Heike Biebermann

**Affiliations:** ^1^Institute of Experimental Pediatric Endocrinology, Charité – Universitätsmedizin Berlin, Freie Universität Berlin, Humboldt-Universitt zu Berlin, Berlin, Germany; ^2^Institute for Community Medicine, University Medicine Greifswald, Greifswald, Germany; ^3^Interfaculty Institute for Genetics and Functional Genomics, University Medicine Greifswald, University of Greifswald, Greifswald, Germany; ^4^Department for Pediatric Endocrinology and Diabetology, Charité – Universitätsmedizin Berlin, Freie Universität Berlin, Humboldt-Universität zu Berlin, Berlin, Germany; ^5^German Center for Diabetes Research (DZD), Greifswald, Germany; ^6^Experimental and Clinical Research Center (ECRC), Charité – Universitätsmedizin Berlin, Max Delbrück Center for Molecular Medicine (HZ), Berlin, Germany; ^7^Institut für Medizinische Physik und Biophysik, Group Protein X-ray Crystallography and Signal Transduction, Charité – Universitätsmedizin Berlin, Freie Universität Berlin, Humboldt-Universität zu Berlin, Berlin, Germany

**Keywords:** trace amine-associated receptor 1, variants, weight regulation, glucose homeostasis, signal transduction

## Abstract

Activation of trace amine-associated receptor 1 (TAAR1) in endocrine pancreas is involved in weight regulation and glucose homeostasis. The purpose of this study was the identification and characterization of potential *TAAR1* variants in patients with overweight/obesity and disturbed glucose homeostasis. Screening for *TAAR1* variants was performed in 314 obese or overweight patients with impaired insulin secretion. The detected variants were functionally characterized concerning TAAR1 cell surface expression and signaling properties and their allele frequencies were determined in the population-based Study of Health in Pomerania (SHIP). Three heterozygous carriers of the single nucleotide missense variants p.Arg23Cys (R23C, rs8192618), p.Ser49Leu (S49L, rs140960896), and p.Ille171Leu (I171L, rs200795344) were detected in the patient cohort. While p.Ser49Leu and p.Ille171Leu were found in obese/overweight patients with slightly impaired glucose homeostasis, p.Arg23Cys was identified in a patient with a complete loss of insulin production. Functional *in vitro* characterization revealed a like wild-type function for I171L, partial loss of function for S49L and a complete loss of function for R23C. The frequency of the R23C variant in 2018 non-diabetic control individuals aged 60 years and older in the general population-based SHIP cohort was lower than in the analyzed patient sample. Both variants are rare in the general population indicating a recent origin in the general gene pool and/or the consequence of pronounced purifying selection, in line with the obvious detrimental effect of the mutations. In conclusion, our study provides hints for the existence of naturally occurring *TAAR1* variants with potential relevance for weight regulation and glucose homeostasis.

## Introduction

The G protein coupled receptor (GPCR) trace amine associated receptor 1 (TAAR1) is activated by biogenic trace amines such as beta-phenylethyl amine (PEA) and tyramine or octopamine (Borowsky et al., 2001; Lindemann and Hoener, 2005). In addition to trace amines, 3-iodothyronamine (T1AM), a molecule with structural similarity to thyroid hormones, is a ligand for rodent and human TAAR1 (Scanlan et al., 2004; Kleinau et al., 2011). So far, no physiological function for the T1AM/TAAR1 complex in humans was described. TAAR1 signals *via* the Gs/adenylyl cyclase system by enhancing intracellular cAMP after ligand challenge.

In pancreatic beta-cells, TAAR1 activation by T1AM enhances insulin secretion (Regard et al., 2007). By using a TAAR1-specific artificial agonist RO5166017 (Revel et al., 2011), the role of TAAR1 in controlling food intake and glucose homeostasis was recently further delineated (Raab et al., 2016). Insulin secretion from a pancreatic beta-cell line and human pancreatic islets could be stimulated under high glucose concentrations (Raab et al., 2016) and treatment of diet-induced obese mice with RO5166017 resulted in reduction of food intake and body weight (Raab et al., 2016). Furthermore, *TAAR1* was found to be highly expressed in pancreatic islets (Regard et al., 2007; Raab et al., 2016), the stomach, and gut (Ito et al., 2009; Revel et al., 2013; Raab et al., 2016).

The relevance of TAAR1 action in human endocrine pancreas physiology needs to be determined since no associations between *TAAR1* variants and obesity or diabetes have been reported to date. Of note, it was recently demonstrated that single nucleotide polymorphisms (SNPs) in human TAAR1 may alter its properties, resulting in expressed, but functional, sub-functional and non-functional receptors, which is of importance for identifying predispositions to human diseases (Shi et al., 2016). In this present study, 314 patients with different levels of impaired glucose homeostasis were screened for *TAAR1* variants. Our data support a potential role of the TAAR1 in glucose homeostasis and body weight regulation in which *TAAR1* variants may predispose to disturbances in maintaining intact insulin secretion.

## Materials and Methods

### Description of the Patient Cohort

Screening for mutations was performed in a group of 314 patients from the Charité Pediatric Diabetes and Obesity outpatient clinic. Of these, 94 patients suffered from antibody-negative diabetes with onset before the age of 18 years and did not have mutations in genes causing monogenic diabetes (*GCK, HNF4A, HNF1A, HNF1B, ABCC8, KCNJ11, INS*). The remaining 220 patients were overweight or obese and exhibited impaired glucose homeostasis. Overweight was defined as >90. percentile and obesity >97. percentile according to German references (Kromeyer-Hauschild et al., 2001). Impaired glucose homeostasis was diagnosed by an increased insulin resistance in a fasting state, determined by elevated HOMA-IR (homeostatic model assessment in insulin resistance) or by an impaired oral glucose tolerance test.

### Description of the Population-Based Control Cohort

The variant frequencies of p.ArgR23Cys (rs8192618) and p.Ser49Leu (rs140960896) were determined for 2,018 diabetes-free participants older than 60 years of age from the population-based SHIP and SHIP-TREND cohorts. Individuals were classified as diabetics if they were diagnosed accordingly (type I or II) by a physician (self-reported during an interview), were under anti-diabetic medication (ATC-code A10), or exhibited increased blood or serum glucose levels (HbA1c ≥ 6.5 and % ≥ 11.1 mmol/l, respectively). All other individuals were classified as diabetes-free.

Study of Health in Pomerania is a population-based project in West Pomerania, a region in the northeast of Germany, that consists of two independent prospectively collected cohorts (SHIP and SHIP-TREND) assessing the prevalence and incidence of common population-based diseases and their risk factors. The study design has been previously described in detail (Volzke et al., 2011).

For SHIP, baseline examinations were carried out from 1997 until 2001, and the sample finally comprised 4,308 participants. Baseline examinations for SHIP-TREND were carried out between 2008 and 2012 and comprised a total of 4420 participants.

### Genotyping of the SHIP and SHIP-TREND Cohorts

The SHIP and SHIP-TREND samples were genotyped using the Illumina ExomeChip array (version Infinium HumanExome v1.0 DNA Analysis BeadChip). Array processing was performed in accordance with the manufacturer’s standard recommendations at the Helmholtz Zentrum München. Genotype calling was performed according to the ExomeChip quality control SOP version 5. Initial genotypes were determined using the GenomeStudio Genotyping Module v1.0 (GenCall algorithm) with the HumanExome-12v1_A manifest file and the standard Illumina cluster file (HumanExome-12v1.egt). Contaminated samples, samples with a call rate < 90%, extreme heterozygosity, extensive estimated IBD sharing with a large number of samples, or mismatch between reported and genotyped sex were excluded. Next, missing genotypes were recalled with the zCall software version 3.3 using default values. In both cohorts, a total of 8,177 individuals were successfully genotyped with an average call rate of 99.97%.

### Variant Screening and Cloning of *TAAR1* Wild-Type and Receptor Variants

PCR amplification of the *TAAR1* gene was performed by using the following primer pairs: forward tttcctcctaggtttctggga and reverse tccaccactgaacagctgac, located in the 5′ and 3′ region of the gene and giving rise to a 1457 bp long PCR fragment. Variant screening was performed by using the amplification primer and two additional primers (CTGGAGCTAAACTTCAAAGG and CTTGCCTGTTCTTTAGCGAT, located in the coding region of the gene) to cover the whole region in both directions using. For sequencing BigDye terminator (PerkinElmer Inc., Waltham, MA, United States) automatic sequencing (ABI3710xl, Applied Biosystems, Foster City, CA, United States) of the coding region of *TAAR1* (NP_612200.1, NM_138327.2) was applied. *TAAR1* wild-type (*TAAR1*-WT) was cloned in the eukaryotic expression vector pcDps as previously described (Kleinau et al., 2011).

The three single nucleotide variants identified by patient screening, p.Arg23Cys (R23C), p.Ser49Leu (S49L), and p.Ille171Leu (I171L), were introduced into *TAAR1* using site-directed mutagenesis according to manufacturer’s instructions of Pfu turbo DNA polymerase (Stratagene, La Jolla, CA, United States).

To enhance cell surface expression for functional analysis, TAAR1 was N-terminally fused with the first 20 amino acids of the bovine rhodopsin (Rho-tag) as previously described (Dinter et al., 2015).

For surface ELISA (enzyme-linked immunosorbent assay), receptor constructs were N-terminally tagged with a hemagglutinin epitope (HA, 5′ YPYDVPDYA 3′) (previous to the Rho-tag).

For SNAP-tag technology, TAAR1-WT and variants were subcloned into the N-terminally SNAP-tagged pcDNA5 vector (New England Biolabs GmbH, Frankfurt am Main, Germany).

### Cell Culture and Transfection

For cAMP assay and SNAP-tag experiments, HEK293 cells were cultured in Minimum Essential Media (MEM) and for cell surface ELISA, COS-7 cells were cultured in Dulbecco’s Modified Eagle Medium (DMEM) (both Biochrom AG, Berlin, Germany), at 37°C and 5% CO_2_. MEM was supplemented with 5% FBS and non-essential amino acids (Biochrom AG, Berlin, Germany). DMEM was supplemented with 5% FBS and 100 U/ml penicillin, 100 μg/ml streptomycin (Biochrom AG, Berlin, Germany), and 2 mM L-glutamine (Invitrogen, Carlsbad, CA, United States). We used two different cell lines because HEK293 cells are an established cell system for investigation of GPCR structure-function relationships. However, for methods that involve intensive washing, like the sandwich ELISA approach, a more robust cell line like COS-7 cells is appropriate.

Transient transfection was carried out using Metafectene (Biontex, Munich, Germany) for cAMP Assay and surface ELISA or FuGene6 (Promega, Fitchburg, WI, United States) for SNAP-tag experiments according to the manufacturers’ protocol.

### Cell Surface Expression Using SNAP-Tag Technology and ELISA

For determination of cell surface expression with fluorescent-based SNAP-tag technology, cells expressing TAAR1-WT and mutant receptor constructs were fixed with 4% PFA and 200 mM HEPES followed by staining with the photostable fluorescent SNAP-Surface^®^ Alexa Fluor^®^ 488 (1:1,000 diluted in Advanced MEM (New England Biolabs GmbH, Frankfurt am Main, Germany), which covalently binds to the SNAP-tag (Cole, 2013).

For SNAP-tag technology nuclei were stained using DAPI dye (Life Technologies, Darmstadt, Germany). Microscopy was performed using an inverted light microscope Axiovert 10 (Carl Zeiss Microscopy GmbH, Oberkochen, Germany) at 63x magnification. Additionally, for verification and quantification of results, a surface ELISA was performed. Therefore, COS-7 cells were transiently transfected with the HA-tagged TAAR1 constructs and empty vector (mock, negative control).

For cell surface expression, cells were fixed with 4% paraformaldehyde and blocked with 10% FBS overnight 48 h after transfection, Next, cells were incubated with biotin-labeled anti-HA antibody (Roche, Basel, Switzerland) (1:200). Bound antibodies were detected with horseradish peroxidase-labeled Streptavidin (BioLegend, London, United Kingdom) (1:2,500). Color reaction was performed as described (Muhlhaus et al., 2014) and absorption was measured at 492 nm and 620 nm using an Anthos Reader 2001 (Anthos Labtech Instruments, Salzburg, Austria).

### Measurement of Gs Activation by cAMP Accumulation Assay Based on AlphaScreen Technology

HEK293 cells transiently expressing empty vector (mock), TAAR1-WT, TAAR1-R23C, TAAR1-S49L, or TAAR1-I171L were left untreated or were stimulated with increasing concentrations of T1AM (from 1 nM to 10 μM) (Santa Cruz Biotechnology Inc., Dallas, TX, United States) or β-phenylethylamine (PEA) 10 μM in the presence of isobuthylmethylxanthine (IBMX). After cell lysis, cAMP levels were measured using AlphaScreen Technology (Perkin-Elmer Life Science, Boston, MA, United States) (Kleinau et al., 2011).

### Statistical Analysis

Statistical analyses were performed using the R statistical software version 2.15.3^1^ and GraphPad Prism 6.0. One-way ANOVA was performed for the effect of TAAR1 stimulation by T1AM or PEA and for cell surface expression.

### Molecular Homology Modeling of TAAR1 in an Inactive State Conformation

The details of structural TAAR1 homology modeling were described previously (Kleinau et al., 2011). In brief, the already determined structure of the β-adrenergic receptor 2 (pdb entry 2RH1, Cherezov et al., 2007) was used as a structural template for TAAR1 modeling in an inactive state, based on high sequence similarity between hTAAR1 and the β-adrenergic receptor 2. The loops were manually adjusted in length and amino acid composition. The ligand bound to the template was deleted. For all modeling procedures, the software package Sybyl 8.0 (Certara, NJ, United States) was used. Substituted side chains and loops were subjected to molecular dynamics simulation (4 ns) by fixing the backbone of the transmembrane helices, followed by conjugate gradient minimizations until converging at a termination gradient of 0.05 kcal/(mol^∗^Å). The AMBER 7.0 force field was used. Structure images were produced using PyMOL Molecular Graphics System, version 1.5, Schrödinger, LLC.

## Results

### Three Heterozygous *TAAR1* Single Nucleotide Variants in Patients with Impaired Glucose Tolerance

Screening of 314 patients with impaired glucose tolerance revealed three heterozygous carriers of single nucleotide variants in the *TAAR1* coding region: p.Arg23Cys (R23C, rs8192618), p.Ser49Leu (S49L, rs140960896), and p.Ile171Leu (I171L, rs200795344). This corresponds to a minor allele frequency (MAF) of 0.16% for each variant in this patient sample.

### Identified TAAR1 Variants Are Properly Expressed at the Cell Surface

**FIGURE 1:**
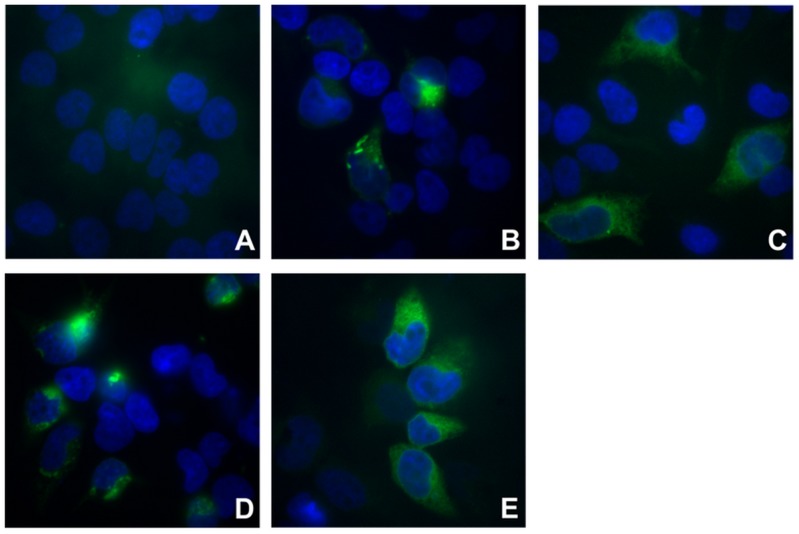
Cell surface expression visualized with fluorescence-based SNAP-tag technology. For fluorescence-based determination of cell surface expression, HEK293 cells expressing empty vector (mock), TAAR1-WT or mutant receptor constructs (R23C, S49L, I171L) were stained with the photostable fluorescent SNAP-Surface^®^ Alexa Fluor^®^ 488 (green). Nuclei were DAPI-stained (blue). Microscopy was carried out with an inverted light microscope Axiovert 10 at 63x magnification. **(A)** mock-transfected cells; **(B)** TAAR1-WT; **(C)** TAAR1-R23C; **(D)** TAAR1-S49L; **(E)** TAAR1-I171L.

**FIGURE 2:**
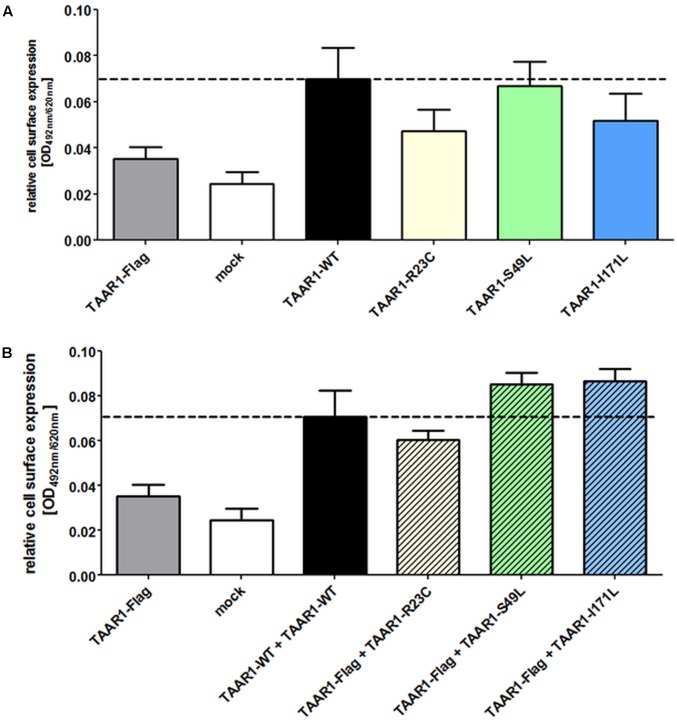
TAAR1 variants show surface expression comparable to TAAR1 wild-type. Cell surface expression of TAAR1 wild-type (TAAR1-WT) and TAAR1 variants was determined via ELISA in COS-7 cells. TAAR1-WT-Flag or empty vector (mock) served as a negative control. Data are depicted as mean ± SEM of relative cell surface expression (OD 492 nm/620 nm) based on three independent experiments performed in four replicates. **(A)** Cells were transiently transfected with HA-tagged TAAR1-WT, TAAR1-R23C, TAAR1-S49L, and TAAR1-I171L. **(B)** For determination of whether cell surface expression of the wild-type receptor is influenced by the mutated receptors, cells were co-transfected with Flag-tagged TAAR1 wild-type and HA-tagged TAAR1-varaints. Statistical analysis was performed using a one-way ANOVA with Kruskal–Wallis test. TAAR1-WT was tested against variants for **(A)** and TAAR1-WT + TAAR1-WT against variants + TAAR1-WT for **(B)**. For **(A,B)** no statistical significant difference was observed.

Two different methods were used to investigate cell surface localization of TAAR1. The SNAP technology revealed TAAR1 wild-type (TAAR1-WT) and variants in intact cells (**Figure 1**), whereas the cell surface ELISA allowed for the quantification of cell surface expression (**Figure 2A**). Using both methods, cell surface localization of all three mutated TAAR1 proteins was statistically not different to wild-type.

### Specific TAAR1 Variants Are Reduced in Signaling Properties

**FIGURE 3:**
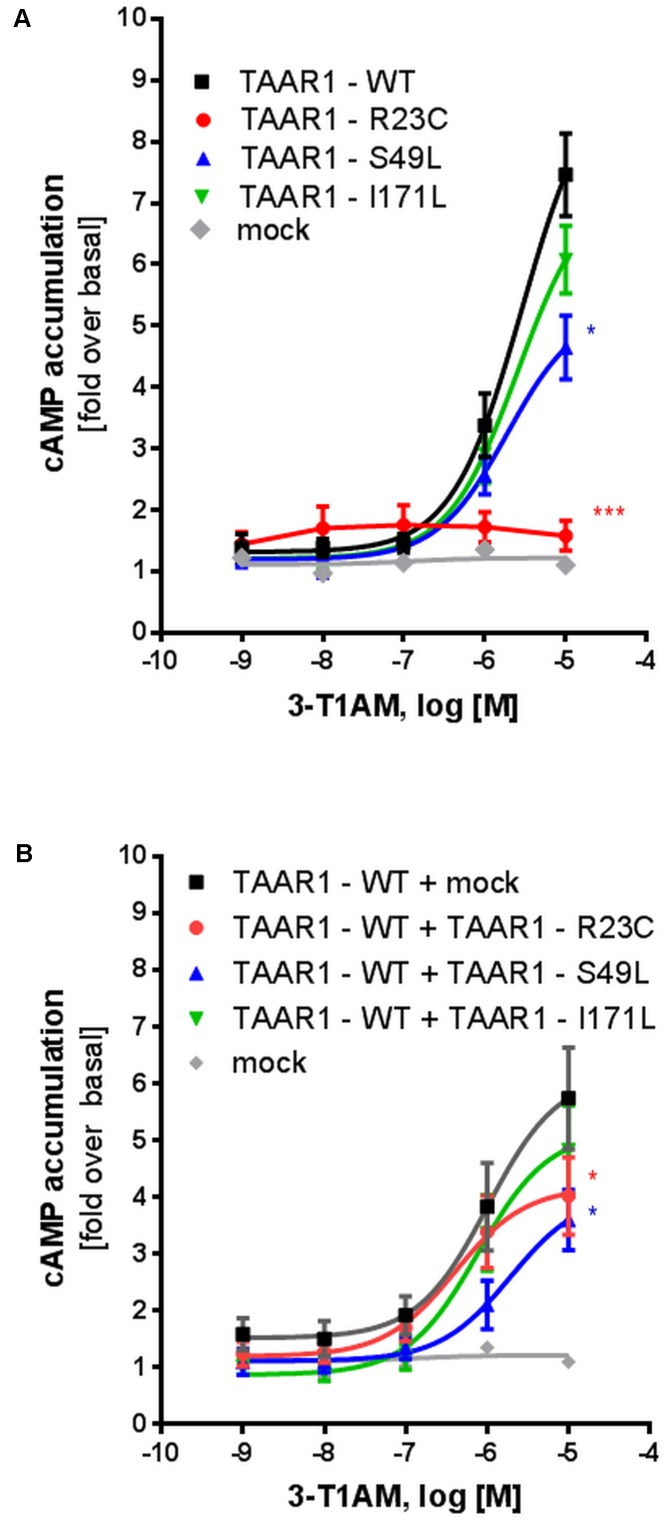
Gs signaling properties of TAAR1 wild-type and TAAR1 variants R23C, S49L, and I171L after stimulation with T1AM. For determination of Gs signaling properties, HEK293 cells, transiently transfected with TAAR1 wild-type (TAAR1-WT) and TAAR1 variants, were stimulated with T1AM (10^-9^M – 10^-5^M) and cAMP accumulation was measured with the AlphaScreen technology. Cells transfected with empty vector (mock) served as a negative control. Data are represented as mean ± SEM of fold over basal of the respective receptor variant and are based on 5–7 independent experiments performed in triplicates. Significance was calculated using one-way ANOVA; ^∗^*p* ≤ 0.05, ^∗∗∗^*p* ≤ 0.001. To mimic the **(A)** homozygous state and the **(B)** heterozygous state, cells were transfected with TAAR1-WT, TAAR1-R23C, TAAR1-S49L, or TAAR1-I171L.

Wild-type TAAR1 and the three TAAR1 mutant proteins were expressed in HEK293 cells and T1AM-induced cAMP accumulation was measured. Compared with wild-type TAAR1 (EC_50_: 2.8 μM), the R23C variant demonstrated a complete loss of function in cAMP accumulation (significant, *p* ≤ 0.001), which did not allow for a proper EC_50_ calculation. Maximal stimulation for S49L and I171L was reduced to 60% (significant, *p* ≤ 0.05) and 80% (not significant) compared with the wild-type, respectively (**Figure 3A**).

To mimic the heterozygous genotype of the patients, co-transfection studies of wild-type (WT) and mutant receptors were performed. For determination of whether cell surface localization of the WT receptor is influenced by the mutated receptors, cells were co-transfected with Flag-tagged *TAAR1*-WT and HA-tagged *TAAR1* variants. While cell surface localization was not impaired for all three variants (**Figure 2B**), R23C and S49L co-expression with TAAR1-WT significantly reduced maximal Gs signaling to 58% (significant, *p* ≤ 0.05), and 55% (significant, *p* ≤ 0.05) of WT + empty vector signaling, respectively (**Figure 3B**), indicating a dominant-negative effect. In contrast, signaling of the TAAR1 I177L variant + TAAR1-WT was not affected. Furthermore, the S49L variant resulted in a slight shift toward higher EC_50_ values (0.7 μM for TAAR1 + empty vector and 1.9 μM for wild-type + S49L) (**Figure 3B**).

**FIGURE 4:**
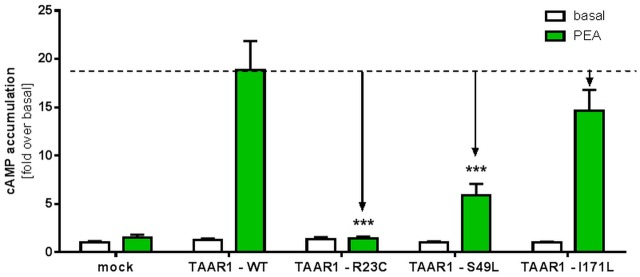
Gs signaling properties of TAAR1 wild-type and TAAR1 variants after stimulation with PEA. TAAR1 wild-type (TAAR1-WT) and TAAR1 variants were stimulated with 10^-5^M agonist beta-phenethylamine (PEA) and cAMP accumulation was measured with the AlphaScreen technology. Cells transfected with empty vector (mock) served as a negative control. Data are represented as mean ± SEM of fold over the basal of the respective receptor variant and are based on four independent experiments performed in triplicates. Significance was calculated using one-way ANOVA; ^∗∗∗^*p* ≤ 0.001.

Beta-phenylethylamine (PEA) is known to be one of the most potent ligands for TAAR1 (Wainscott et al., 2007). For determination of Gs signaling properties after stimulation with PEA, HEK293 cells transiently transfected with *TAAR1* wild-type (*TAAR1*-WT) and *TAAR1* variants were stimulated with 10^-5^M PEA and cAMP accumulation was measured. The R23C mutant showed a complete loss of function indicated by non-responsiveness to PEA (significant, *p* ≤ 0.001) (**Figure 4**). The Gs/adenylyl cyclase-mediated signaling of the S49L variant demonstrated a significant signal reduction of approximately 70%, (*p* ≤ 0.001) while the I171L substitution exhibited only a slight, but not significant, reduction of cAMP accumulation.

In summary, R23C represented a complete loss-of-function variant, S49L a partial loss-of-function variant, and despite a slightly reduced maximal signaling capacity, I171L was classified as WT-like and excluded from further analysis.

### Patient Phenotypes

*Patient 1* (variant p.Arg23Cys carrier) was born small for gestational age to non-related parents of Middle Eastern background. He developed insulin-dependent diabetes at 3 years of age with low C-peptide, and negative anti-GAD and anti-insulin antibodies. Further evaluation did not reveal variants in known monogenic diabetes genes. At 7 years of age, his mental performance was tested and revealed an IQ of 71. Additionally, his growth rate consistently decreased and at 6 years of age, he was tested for growth hormone deficiency with normal MRI findings of the brain and pituitary gland. His father (non-carrier of a variant) developed type 2 diabetes at 40 years of age and was not obese. Treatment was comprised of oral antidiabetic drugs. The mother is a heterozygous variant carrier and is normoglycemic. Thyroid hormone levels were in the reference range.

*Patient 2* (variant p.Ile171Leu carrier) was diagnosed at 11 4/12 years of age with obesity (BMI, 30.0 kg/m^2^; +2.59 BMI-SDS) and a disturbed glucose homeostasis (HOMA-IR, 2.8) and transient pathological oral glucose tolerance test. There was no family background of type 2 diabetes or cardiovascular problems. Lifestyle intervention resulted in weight loss (BMI, 25.5 kg/m^2^) and HOMA-IR values normalized but were not completely stable (0.7–1.7). Thyroid function was normal.

*Patient 3* (variant p.Ser49Leu carrier) was overweight (BMI 25.0 kg/m^2^) and had a disturbed glucose homeostasis (IGT; HOMA-IR, 2.9). Lifestyle intervention resulted in weight loss and normalization of HOMA-IR; however, the patient regained weight (BMI, 29.0 kg/m^2^) and HOMA-IR increased to 6. There was no reported family history of type 2 diabetes or cardiovascular complications. Thyroid function was normal. The patient discontinued from the treatment group because of psychiatric problems.

Samples from the parents of patients 2 and 3 were unavailable.

### Variant Frequencies in a Large Control Population

The results so far suggest that two of the three single nucleotide variants, namely p.Arg23Cys (rs8192618) and p.Ser49Leu (rs140960896), might predispose to impaired glucose homeostasis. The design of a proper control cohort to detect putative enrichment of these variants in patients had to anticipate a variable and mild manifestation of the phenotype with increasing age. Therefore, participants of the SHIP study that were ≥60 years of age and diabetes-free were assigned to this control group, thereby increasing the likelihood of including unaffected individuals. Within the SHIP and SHIP-TREND cohorts, 2,018 individuals that were ≥60 years of age were diagnosed as diabetes-free (see description of the control population) and were subsequently analyzed for the p.Arg23Cys (rs8192618, genotyping data for 2,009 individuals available) and p.Ser49Leu (rs140960896, genotyping data for 2,018 individuals available) allele frequencies. In this control group, only nine individuals were found to be heterozygous carriers of p.Ser49Leu, while no carriers were observed for p.Arg23Cys (**Table 1**). In the remaining SHIP sample, patients with <60 years of age that included individuals with the potential of developing diabetes at an older age, the number of heterozygous p.Ser49Leu carriers amounted to 18 of 5,149 individuals with available genotyping data. Four heterozygous p.Arg23Cys carriers were found among the 5,163 individuals with the available genotyping data. The corresponding single nucleotide variant minor allele frequencies (MAFs) for the older control cohort, the younger remaining cohort, and the total SHIP study were 0.00%, 0.04%, and 0.03% for p.Arg23Cys (rs8192618), respectively, and 0.22%, 0.17%, and 0.19% for p.Ser49Leu (rs140960896), respectively. The MAFs reported for the aggregated general population (121,408 samples from the dbSNP database^2^) amount to 0.154 and 0.038% for p.Arg23Cys and p.Ser49Leu, respectively.

**Table 1:** Genotype frequencies of the *TAAR1* variants p.Arg23Cys and p.Ser49Leu of diabetes-free individuals from the population-based SHIP-cohort.

	p.Ser49Leu (S49L, rs140960896)			p.Arg23Cys (R23C, rs8192618)		
		
Age group (years) [*n*]	Non-carriers [*n*]	Heterozygote A alleles [*n*]	MAF (%)	Non-carriers [*n*]	Heterozygote A alleles [*n*]	MAF (%)
20–29	889	2	0.11%	890	1	0.06%
30–39	1,356	6	0.22%	1,361	1	0.04%
40–49	1,457	3	0.10%	1,459	1	0.03%
50–59	1,447	7	0.24%	1,453	1	0.03%
*Total (younger)*	*5,149*	*18*	*0.17%*	*5,163*	*4*	*0.04%*
						
60–69	1,207	5	0.21%	1,212	0	0.00%
70–79	738	4	0.27%	742	0	0.00%
80–83	64	0	0.00%	64	0	0.00%
*Total (older)*	*2,009*	*9*	*0.22%*	*2,018*	*0*	*0.00%*
***Overall***	***7,158***	***27***	***0.19%***	***7,181***	***4***	***0.03%***


*The genotype frequencies of the single nucleotide variants p.Arg23Cys (R23C, rs8192618) and p.Ser49Leu (S49L, rs140960896) are shown for 5,167 diabetes-free SHIP and SHIP-TREND participants aged younger than 60 years of age (upper part) and 2,018 individuals aged 60 years and older (lower part). MAF, minor allele frequency.*

## Discussion

Recently, a physiological role for TAAR1 in glucose homeostasis and food intake in rodents was reported (Raab et al., 2016). Due to the generation of a new monoclonal antibody, the expression spectrum of human *TAAR1* was reported in endocrine organs that are capable of secreting hormonal signals involved in energy metabolism such as the pancreas, stomach, and gut (Raab et al., 2016). Of specific note, stimulation of a rat pancreatic islet cell line or human isolated pancreatic islets with the TAAR1 specific agonist RO5166017 led to an increase in insulin secretion and application of RO5166017 to diet-induced obese mice reduced food intake and body weight (Raab et al., 2016). Yet, the pathophysiological role of TAAR1 action in humans remains elusive. In the present study, we screened for *TAAR1* variants in a cohort of patients that were overweight/obese with disturbed glucose homeostasis and in patients with a lack of appropriate insulin secretion. Several genes are known to cause monogenic obesity (Farooqi and O’Rahilly, 2008) and diabetes (Henzen, 2012); however, variations in the genes of polygenic obesity also add to the complex clinical spectrum (Hinney et al., 2010; van der Klaauw and Farooqi, 2015).

Two *TAAR1* variants, namely, p.Arg23Cys (rs8192618) and p.Ser49Leu (rs140960896), were identified in the currently investigated patient sample. Data from the population-based SHIP study were used to determine the frequencies of these variants in control individuals recruited from the general adult population. To optimize the control cohort to comprise of predominantly healthy individuals, we assigned diabetes-free SHIP participants aged 60 years and older to this group. In contrast to the patient sample, no carrier of the p.Arg23Cys variant was observed in this cohort, while nine heterozygous carriers of the p.Ser49Leu variant were found. The fact that the frequency differences of the two variants between the defined control cohort and the patient sample did not show statistical significance is mainly explained by the low frequencies of p.Arg23Cys and p.Ser49Leu in the general population. Therefore, larger study samples would be required to validate the observed associations with the disease phenotypes. For example, data from approximately 700–1,000 patients are required to reveal a statistically significant (*P* < 0.05) difference in the allele distribution between patients and diabetes-free controls with an 80% power given to the observed p.Arg23Cys minor allele frequency (MAF) of 0.03% in the SHIP cohort. The other putative risk variant p.Ser49Leu exhibited a MAF of 0.19% in the total analyzed SHIP cohort. In the dbSNP database based on genotyping data of 121,408 samples, MAFs of 0.15, 0.04, and 0.02% were reported for p.Arg23Cys, p.Ser49Leu and p.Ile171Leu, respectively. In spite of these MAF differences, the two variants in any case belong to the category of low frequency variants (MAF ≤ 0.5%) (The 1000 Genomes Project Consortium, 2015), indicating their recent origin in the general gene pool and/or the consequence of pronounced purifying selection, in line with the predicted detrimental effect of the resulting amino acid exchanges.

Recently, a screen for *TAAR1* variants in patients suffering from schizophrenia was performed. In this study they identified the variant p.Cys182Phe in three affected members of a family. Prediction tools indicated that this variant might be of functional relevance. In addition, by screening more patients the authors identified six additional variants of which probably four were of functional relevance (John et al., 2017). Here functional studies may provide additional evidence if these variants are associated with the phenotype of the patients.

In our study, functional TAAR1 characterization, including determination of cell surface localization and signaling properties (Gs), revealed a complete loss of function for the R23C variant. While the heterozygous variant carrier lacked insulin secretion, his mother exhibited normal Hb1C levels in spite of the same genotype. The father of the patient was not a *TAAR1* variant carrier; however, he suffered from type 2 diabetes of unknown origin. Therefore, in addition to the maternally inherited p.Arg23Cys variant, at least one additional (unknown) diabetes-predisposing variant may have been potentially inherited from the father accounting for the patient’s phenotype. Such an effect was already demonstrated for variants involved in congenital hypothyroidism (Sriphrapradang et al., 2011).

Interestingly, functional characterization of S49L, especially in the heterozygous state, decreases TAAR1 signaling induced by T1AM or PEA (**Figures 3A**, **4**). Consistently, the corresponding patient was found to be greatly affected compared with the patient carrying the I171L variant.

**FIGURE 5:**
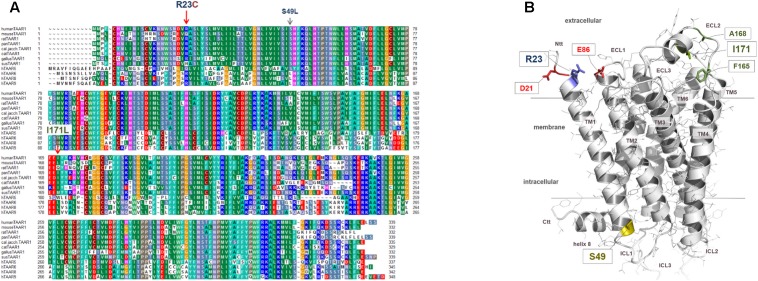
Identified TAAR1 sequence variations mapped to a three-dimensional TAAR1 model in an inactive conformation. **(A)** This sequence alignment shows overlapping or diverse amino acids at corresponding positions between several TAAR1 orthologous and additionally compared to other human TAAR group members (alignment is performed manually; the alignment was visualized with the software BioEdit.). The positions of the here investigated sequence variants are indicated by arrows. Particular background colors, indicating conservation (Blossum62 matrix) among different receptors and simultaneously reflecting biophysical properties of the amino acid side chains: black – proline, blue – positively charged, cyan/green – aromatic and hydrophobic, green – hydrophobic, red – negatively charged, gray – hydrophilic, dark-red – cysteines, magenta – histidine. **(B)** The 3-dimensional hTAAR1 model shows the spatial localization of the wild-type amino acids (colored sticks, labeled) at the positions of identified substitutions. The model is based on a previously described modeling approach (Kleinau et al., 2011). Arginine 23 is located at the transition between transmembrane helix 1 (TM1) and the N-terminal tail (Ntt) at the extracellular side. The side chain of Arg23 can potentially interact with residues of the extracellular loop 1 (ECL1) such as the negatively charged Glu86 or with the Asp21 located in the N-terminal tail (Ntt). The amino acid Arg23 is conserved among TAAR1 orthologous (two variants Qln, His). In contrast, Ser49 points to the cytosol at the transition between TM1 and the intracellular loop 1 (ICL1). This residue is highly conserved throughout TAAR1 orthologs (see **A**) and may be involved in the maintenance of structural conformation in this specific receptor part, whereby a direct contact partner cannot be observed in our model. Isoleucine at position 171 is located in the putative helical extracellular loop 2 (ECL2) structure and the side chain is close to hydrophobic side chains of amino acids like Ala168 or Phe165.

The arginine residue at position 23 of TAAR1 is located at the transition between transmembrane helix 1 (TM1) and the N-terminal tail (Ntt) at the extracellular side of the receptor (**Figure 5**). The side chain of Arg23 may interact with residues of the extracellular loop 1 such as the negatively charged Glu86 or the Asp21 at the N-terminus. Both amino acids are conserved among TAAR1 orthologous, whereby Glu86 - in contrast to Asp21- is also conserved among different human TAAR subtypes, which supposes in combination with the high conservation of Arg23 an interaction between these two residues. In contrast, serine 49 points to the cytosol at the transition between TM1 and the intracellular loop 1. This residue is highly conserved throughout TAAR1 orthologous (but not in the TAAR group) and may be involved in the maintenance of the structural conformation in this specific receptor section by a H-bond to the helical backbone. Modifications at this position should therefore lead to decreased capacity of the receptor to interact with the G-protein.

In summary, we identified *TAAR1* variants in a patient cohort affected by overweight/obesity and disturbed glucose homeostasis. Functional characterization of the corresponding protein variants revealed substantial effects of R23C and S49L on receptor signaling. We observed only a trend toward a higher frequency of these variants in our patient cohort compared with a population-based control sample, which, however, may be explained by a lack of power due to the low frequencies of the variants in the general population. Therefore, additional studies in larger cohorts are necessary to further unravel the complete potential of TAAR1 in human beta-cell function. So far, we conclude that *TAAR1* variants may represent at least low frequent modifiers of insulin secretion, glucose homeostasis, and weight regulation.

## Ethics Statement

Ethical approval for all studies was obtained from the Charité ethical committee (EA2/077/06). Informed consent was obtained from all participants or their parents/legal guardians, and institutional review approval was received for this study. The medical ethics committee of the University of Greifswald approved the study protocol and oral and written informed consents were obtained from each study participant. This study was conducted in accordance with the declaration of Helsinki.

## Author Contributions

JM: experimental design, performed the experiments, data analysis, evaluation and discussion, wrote the manuscript and final approval. JD: experimental design, performed the experiments, data analysis, evaluation and discussion, final approval. SJ: mutational screening, performed SNAP-Tag data, figure preparation, final approval. AT: performed epidemiological data, data analysis, wrote the manuscript, final approval. SFJ: mutational screening, patient selection, data evaluation, final approval. GH: performed epidemiological data, data analysis, wrote the manuscript, final approval. PK: mutational screening, patient selection, data evaluation, final approval. SW: mutational screening, patient selection, data evaluation, final approval. AG: patient selection, data evaluation, final approval. HV: performed epidemiological data, data analysis, wrote the manuscript, final approval. KR: mutational screening, patient selection, data evaluation, final approval. GK: experimental design, modeling of TAAR1 mutations, performed the experiments, data analysis, evaluation and discussion, wrote the manuscript and final approval. HK: patient selection, data analysis, evaluation and discussion, wrote the manuscript and final approval. HB: study design, performed the experiments, data analysis, evaluation and discussion, wrote the manuscript and final approval.

## Conflict of Interest Statement

The authors declare that the research was conducted in the absence of any commercial or financial relationships that could be construed as a potential conflict of interest.

## Footnotes

1 www.R-project.org

2 http://www.ncbi.nlm.nih.gov/projects/SNP/
